# Photon-counting CT in maxillofacial and temporal bone CT—a comparative analysis of image quality and dose with high-end energy-integrating CT systems

**DOI:** 10.1186/s41747-025-00618-6

**Published:** 2025-08-15

**Authors:** Yannik Christian Layer, Narine Mesropyan, Alexander Isaak, Dmitrij Kravchenko, Leon Bischoff, Claus C. Pieper, Patrick Kupczyk, Julian A. Luetkens, Benjamin P. Ernst, Daniel Kuetting

**Affiliations:** 1 Department of Diagnostic and Interventional Radiology, University Hospital Bonn, Bonn, Germany; 2 Quantitative Imaging Lab Bonn (QILaB), Bonn, Germany; 3 Department of Otorhinolaryngology, University Hospital Frankfurt, Frankfurt, Germany

**Keywords:** Computed tomography, Dose reduction, Maxillofacial CT, Photon-counting detector CT, Temporal bone

## Abstract

**Background:**

This experimental study aimed to compare the image quality of maxillofacial and temporal bone imaging using different radiation dose settings on current high-end CT systems: photon-counting detector CT (PCDCT), dual-source energy-integrating detector CT (DECT), and dual-layer spectral detector CT (SDCT).

**Materials and methods:**

CT scans of a cadaveric human specimen were investigated. Temporal bone imaging was performed with the following parameters: 120 kV and **A** (high-dose): 140–100 mAs; **B** (middle-dose): 90–60 mAs; **C** (low-dose): 50–25 mAs; **D** (ultra-low-dose): 20–10 mAs. Similarly, for maxillofacial CT: 100 kV and **A**: 100–80 mAs; **B**: 70–50 mAs; **C**: 40–25 mAs; **D**: 20–10 mAs. Region of interest (ROI)-based noise, SNR, and CNR ratios were calculated for objective assessment of image quality. Subjectively, image quality (IQ) of important anatomic landmarks was assessed using a Likert grading scale from 1 (non-diagnostic) to 5 (excellent).

**Results:**

For temporal bone, PCDCT provided excellent-to-good IQ up to low-dose scans for all anatomical landmarks, which was superior to SDCT (excellent-to-sufficient), followed by DECT (good-to-poor): *e.g.*, for **C**: 4.3 ± 0.5 *versus* 3.7 ± 0.6 *versus* 2.9 ± 0.6, *p* < 0.001. PCDCT had significantly better IQ compared to SDCT in ultra-low-dose settings (**D**: 3.9 ± 0.4 *versus* 2.8 ± 0.4, *p* < 0.001). For maxillofacial CT, no significant differences in IQ were found between all CT systems using high- and middle-dose scans, *e.g.*, **B**: 3.9 ± 0.5 *versus* 3.8 ± 0.7 *versus* 3.8 ± 0.4 (*p* = 0.81). In low- and ultra-low-dose settings, IQ was similar by PCDCT and SDCT (**C**: *p* = 0.17; **D**: *p* = 0.99) and superior to that of DECT (**C**: *p* < 0.05).

**Conclusion:**

PCDCT offers excellent image quality for temporal bone and maxillofacial CT even at ultra-low doses; results were, in some cases, superior to SDCT and DECT.

**Relevance statement:**

As PCDCT outperformed modern DECT and SDCT in assessment of maxillofacial and temporal bone CT for image quality and radiation dose, our study suggests that the implementation of PCDCT will improve image quality while reducing radiation exposure in general population.

**Key Points:**

- This work compares the quality of maxillofacial and temporal bone imaging in PCDCT, DECT, and SDCT.
- Scans of a cadaveric human specimen were investigated using different radiation doses.
- PCDCT offers excellent image quality for temporal bone and maxillofacial CT.
- PCDCT, SDCT, and DECT all showed good image quality overall.

**Graphical Abstract:**

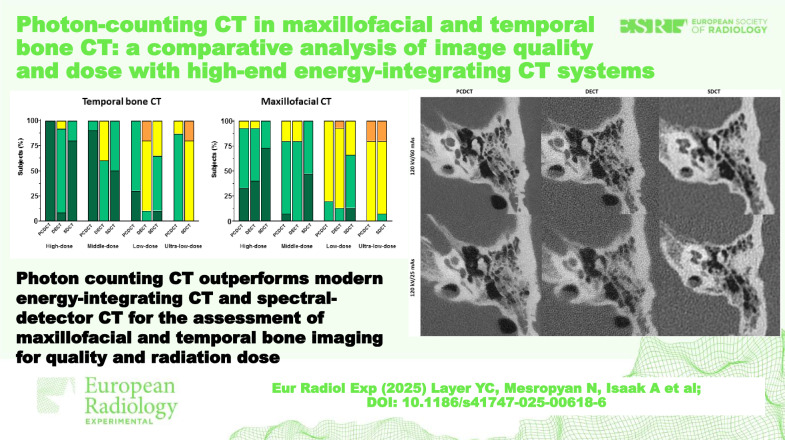

## Introduction

Over the last decades, computed tomography (CT) has undergone significant technological advancements, establishing it as an indispensable diagnostic tool in maxillofacial and temporal bone imaging. As high spatial resolution is a requisite for sufficient imaging of these complex and often miniature anatomical structures (*e.g.,* facial nerve canal, cochlea, tympanic tegmen), high radiation is often applied [1, 2]. This poses a challenge due to the proximity of radiosensitive organs in the scan volume (*e.g.,* eye lenses), especially in the pediatric population, young adults, and patients undergoing repetitive examinations.

Thus, there is a clinical need for further optimization of CT imaging, enabling high spatial imaging with low radiation dose. A number of studies have investigated the potential of low and ultra-low-dose protocols in maxillofacial/temporal bone CT imaging. However, in most previous studies, scanners with energy-integrating detectors (EIDs) were analyzed [3–5]. Other studies on photon-counting detector CT (PCDCT) were missing a comparison with modern high-end CT scanners [6–10]. PCDCT technology offers advantages over EIDs, thus enabling dramatically improved maxillofacial and temporal bone CT imaging [11].

The aim of this experimental study was to investigate the potential of a clinically implemented PCDCT regarding image quality at reduced dose for clinical care in the maxillofacial region and temporal bone imaging using different radiation dose settings in comparison to other currently commercially available state-of-the-art CT systems: Dual-layer spectral detector CT (SDCT) and dual-source dual-energy EID-based CT (DECT).

## Materials and methods

The local institutional review board approved this experimental study (Ethics Committee of the Medical Faculty of the University of Bonn; reference number 187/23-EP). A single cadaveric human head was obtained from the local anatomical institute. During this experimental study all local and international ethical guidelines and laws that pertain to the use of human cadaveric donors in medical research were complied with.

### Computed tomography

**Fig. 1:**
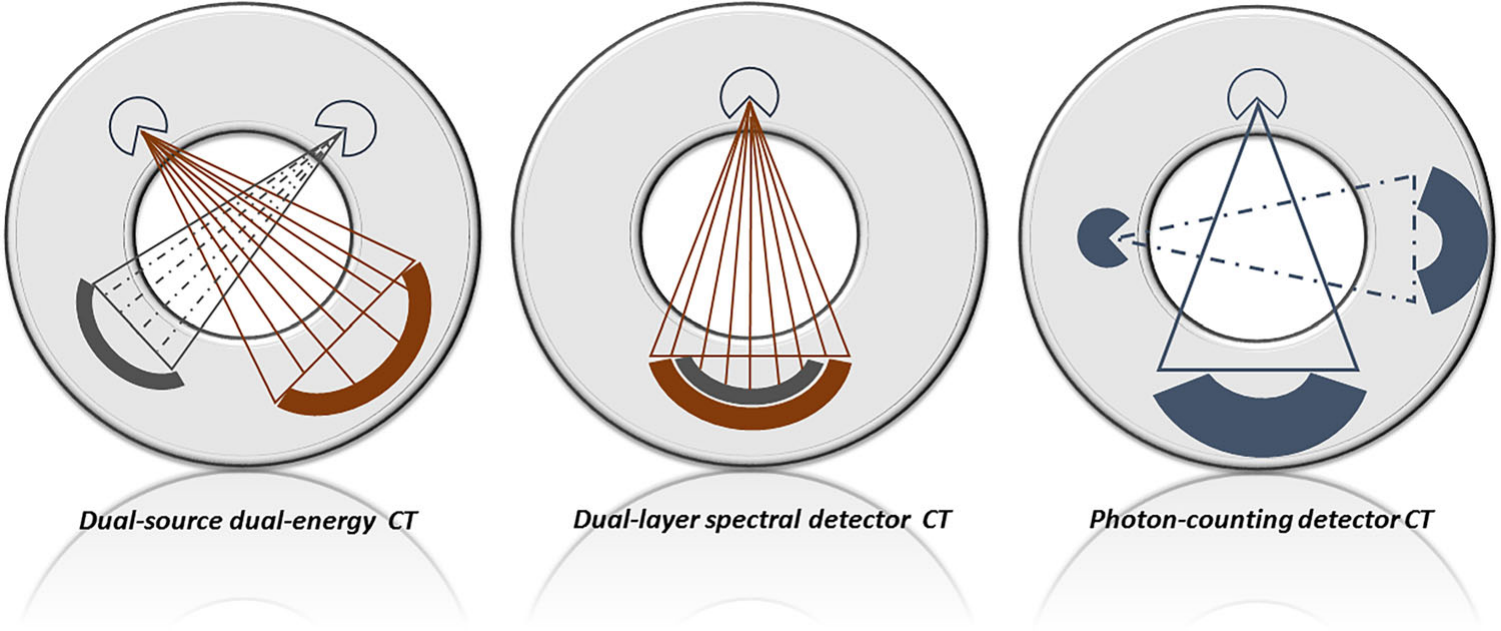
Study design. Three different high-end CT systems were used for this experimental study: Energy-integrating detector (EID)-based dual-energy dual-source CT (DECT), dual-layer spectral detector CT (SDCT) and photon-counting detector CT (PCDCT). DECT uses two x-ray tubes (brown: high-energy tube, gray: low-energy tube) and corresponding detectors (high-energy tube field of view, low-energy field of view). SDCT employs two layers within the detector; the superficial layer (brown) absorbs low-energy x-ray photons, and the deep layer (gray) absorbs high-energy photons. PCDCT is built based on a dual-source CT basis, where the EID arrays are replaced with cadmium telluride-based photon-counting detector arrays. In this study, all CT scanners operated in single-source mode

The maxillofacial and temporal bone regions were scanned separately using three different high-end CT systems: photon-counting detector CT (NAEOTOM Alpha; Siemens Healthcare, Germany), 3rd generation dual-source dual-energy CT (SOMATOM Force; Siemens Healthcare, Germany), and dual-layer spectral detector CT (IQon, Philips Healthcare, The Netherlands) (Fig. 1). All CT examinations were performed in a single-energy mode using predetermined constant tube voltage (kV) of 100 kV for maxillofacial CT and 120 kV for temporal bone CT with different tube current (mAs). For maxillofacial CT following radiation dose settings were employed: 100 kV and high-dose protocols (A): 100 mAs, 90 mAs, 80 mAs; middle-dose protocols (B): 70 mAs, 60 mAs, 50 mAs; low-dose protocols (C): 40 mAs, 30 mAs, 25 mAs. For temporal bone CT energy dose settings were as follows: 120 kV and high-dose protocols (A): 140 mAs, 130 mAs, 120 mAs, 110 mAs, 100 mAs; middle-dose protocols (B): 90 mAs, 80 mAs, 70 mAs, 60 mAs; low-dose protocols (C): 50 mAs, 40 mAs, 30 mAs, 25 mAs. Additionally, maxillofacial and temporal bone CT examinations of cadaveric specimens in ultra-low-dose settings with tube currents of 20 mAs, 15mAs and 10 mAs were performed on PCDCT and SDCT (Protocols D). Scanning in ultra-low-dose setting with tube current under 25 mAs was not feasible on DECT. Collimation was 120 × 0.2 mm in PCDCT, 24 × 0.6 mm in EIDCT and 64 × 0.6 mm in SDCT. All settings were chosen by vendor recommendations using the settings configured in the specific program. Pitch was 0.85 for PCDCT and DECT and 0.296 for SDCT. The scanning protocols and image acquisition parameters (except for the tube current) were identical on all scanners for all examinations. Additionally, vendor-specific tin filtration (Sn) was used for maxillofacial CT in DECT and PCDCT, enabling additional radiation dose reduction. Temporal bone was scanned without tin filtration. No tin filtration was available for the SDCT scanner.

Datasets were reconstructed in a bone and soft tissue window using dedicated temporal bone and maxillofacial imaging sharp kernels for each scanner, respectively (PCDCT: Hr40/ Hr68 and Hr84; DECT: Hr36/Hr54 and Ur77; SDCT: UB/YB and YC) as well as vendor-specific iterative image reconstruction. In this study, iterative reconstruction of DECT (ADMIRE, Siemens Healthcare) was set to strength 4/5, of PCDCT (QIR, Siemens Healthcare) was set to level 3/4 and SDCT (iDose, Philips) was set to level 4/7. A manual setting of 0 for all scanners was not possible, and settings were chosen based on vendor recommendation as per standard technical protocol.

All coronal and axial planes were reformatted with 1.0 mm slice thickness for maxillofacial and 0.6 mm for temporal bone CT.

### Image analysis

All image analysis was performed in consensus by two board-certified radiologists with 10 (D.K.) and 4 (N.M.) years of experience in temporal bone and maxillofacial imaging. Images were presented in random order, and readers were blinded to the acquisition parameters and scanners‘ data. Image analysis was performed using the institutional picture archiving and communication system (Deep Unity R20 XX, Dedalus HealthCare, Germany). For both maxillofacial and temporal bone CT, subjective (qualitative) image quality and objective (quantitative) image quality (noise, SNR and CNR) were evaluated and compared for each CT system. Additionally, dose-length product (DLP) and CT dose index (CTDI) were retrieved for each scan and compared.

### Subjective (qualitative) image quality

Subjective image quality was rated in consensus. Image quality of clinically relevant anatomical landmarks (for maxillofacial: uncinate process, nasolacrimal duct, cribriform plate, maxillary ostium, overall; for temporal bone: facial nerve canal, cochlea, tympanic tegmen, malleus, overall) was separately assessed using 5-point Likert grading: 5 = excellent image quality (excellent delineation with no artifacts resulting in high diagnostic confidence); 4 = good image quality (good delineation with slight artifacts resulting in good diagnostic confidence); 3 = intermediate/sufficient image quality (little blurring, some artifacts resulting in decreased diagnostic confidence); 2 = poor/insufficient image quality (severe blurring and/or artifacts); 1 = non-diagnostic image quality (borders are non-identifiable with severe artifacts). The overall image quality score was calculated for each scan protocol as the mean of individual points. Additionally, the mean image quality score for scans in high-, middle-, low- and ultra-low-dose settings was calculated and used for final analysis.

### Objective (quantitative) image quality

For objective assessment of image quality, region of interest (ROI)-based noise, SNR, and CNR ratios were calculated for temporal bone CT. ROIs were drawn as large as possible with an approximate ROI size of 1 cm^2^ in consensus by both readers (two board-certified radiologists with 10 (D.K.) and 4 (N.M.) years of experience in temporal bone and maxillofacial imaging). For temporal bone CT, ROIs were placed in the retrobulbar fat and in the air anterior to the external ear on both sides. Noise was determined as the standard deviation (SD) of the measurement in each of the ROIs. Each of the measurements was performed twice, and the mean of these measurements was used for the final analysis to ensure measurement consistency. Bone window was used for all measurements. SNR and CNR were calculated using the following equations, where AV is the average attenuation value measured in Hounsfield units (HU):

$${{{{\rm{SNR}}}}}_{{{{\rm{temporal\; bone}}}}}=({{{{\rm{AV}}}}}_{{{{\rm{retrobulbar}}}}\; {{{\rm{fat}}}}}/{{{\rm{AV}}}}_{{{\rm{noise}}}})$$

$${{{{\rm{CNR}}}}}_{{{{\rm{temporal\; bone}}}}}=({{{{\rm{AV}}}}}_{{{{\rm{retrobulbar\; fat}}}}}-{{{{\rm{AV}}}}}_{{{{\rm{noise}}}}}/{{{\rm{AV}}}}_{{{\rm{air}}}})$$

### Statistical analysis

Commercially available software (SPSS Statistics, Version 25, IBM, USA and Prism 9, GraphPad Software, USA) was used for statistical analysis. Data were checked for normal distribution using Shapiro–Wilk test. Data are given as mean ± standard deviation or absolute frequencies, as appropriate. Friedman test followed by Dunn’s multiple comparison test was used to compare the subjective image quality in high-, middle-, and low-dose settings between all evaluated CT systems. The Wilcoxon signed-rank test was used to evaluate the differences regarding image quality in ultra-low-dose settings between SDCT and PCDCT. The level of statistical significance was set to *p* < 0.05.

## Results

A total of 78 CT scans were performed, and 390 assessment points (45 temporal bone scans with 225 assessment points and 33 maxillofacial scans with 165 assessment points) were analyzed.

### Subjective image quality analysis

#### Maxillofacial CT

**Fig. 2:**
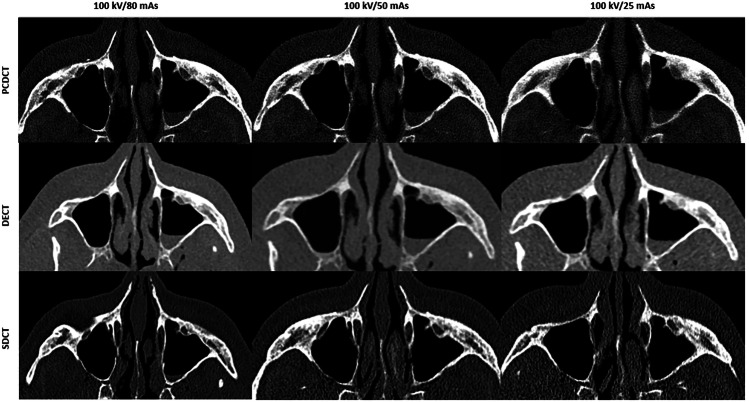
Representative images of maxillofacial CT from photon-counting detector CT (PCDCT) and energy-integrating detector (EID)-based CT systems acquired in high-, middle-, and low-dose scans. The images were reconstructed in bone window. PCDCT provides stable, sufficient for diagnostic use CT images up to low-dose scans by significantly reduced radiation exposure compared to spectral detector CT (SDCT). PCDCT and dual-source, dual-energy (DECT) images are acquired with a tin filter

For the maxillofacial region, PCDCT and DECT (both acquired with tin filter) achieved excellent-to-sufficient image quality in all assessment points in high-dose settings, which was lower than that of SDCT (excellent-to-good) as presented in Table 1. A single sufficient image quality score was rated for PCDCT and DECT for assessment of cribriform plate in 100 kV images acquired with 80 mAs. Although no differences were observed in mean image quality scores for middle-dose settings between all CT systems, PCDCT and DECT provided excellent-to-sufficient image quality, while SDCT provided excellent-to-good image quality. In low-dose settings, PCDCT (good-to-sufficient) and DECT (good-to-poor) showed similar image quality (3.2 ± 0.4 *versus* 3.1 ± 0.5; *p* = 0.184), which was significantly lower than that of SDCT (excellent-to-sufficient) (3.8 ± 0.7; *p* = 0.041). No differences in image quality were observed between SDCT (good-to-poor) and PCDCT (sufficient-to-poor) in ultra-low-dose settings, with scores of 2.8 ± 0.4 and 2.9 ± 0.5, respectively (*p* = 0.422). Representative images of maxillofacial CT using different radiation dose settings in different CT systems are presented in Fig. 2.

**Table 1:** Mean values of image quality scores assessed in consensus for both readers by photon-counting detector CT, dual-source dual-energy CT and dual-layer spectral detector CT using different radiation dose settings

	Photon-counting detector CT	Dual-source dual-energy, energy-integrating detector CT	Dual-layer spectral detector CT
Maxillofacial CT			
High-dose	4.3 ± 0.6	4.3 ± 0.6	**4.7** ± **0.5**
Middle-dose	3.9 ± 0.5	3.8 ± 0.4	3.8 ± 0.7
Low-dose	3.2 ± 0.4	3.1 ± 0.5	**3.8** ± **0.7**
Ultra-low-dose	2.8 ± 0.4	-	2.9 ± 0.5
Temporal bone CT			
High-dose	5.0	**4.0** ± **0.4**	**4.8** ± **0.4**
Middle-dose	4.9 ± 0.3	**3.6** ± **0.5**	**3.8** ± **0.6**
Low-dose	4.3 ± 0.5	**2.9** ± **0.6**	**3.8** ± **0.6**
Ultra-low-dose	3.9 ± 0.4	-	**2.8** ± **0.4**

For maxillofacial CT, high-dose settings included protocols with: 100 mAs, 90 mAs, 80 mAs; middle-dose: 70 mAs, 60 mAs, 50 mAs; low-dose: 40 mAs, 30 mAs, 25 mAs. For temporal bone CT, high-dose protocols included: 140 mAs, 130 mAs, 120 mAs, 110 mAs, 100 mAs; middle-dose: 90 mAs, 80 mAs, 70 mAs, 60 mAs; low-dose: 50 mAs, 40 mAs, 30 mAs, 25 mAs. Mean values of image quality scores assessed in consensus by both readers for all anatomical landmarks are presented. Continuous variables are given as means ± standard deviations. *p*-values were obtained using Wilcoxon signed-rank test. Significant values are marked in bold (*p* < 0.05)

#### Temporal bone CT

Over the range of radiation dose settings studied, PCDCT consistently demonstrated superior image quality compared to DECT and SDCT for temporal bone CT, with excellent-to-good image quality provided up to low-dose scans for all anatomical landmarks. In high-dose protocols, PCDCT produced image quality scores of 5 ± 0, whereas SDCT and DECT achieved scores of 4.8 ± 0.4 (*p* = 0.004) and 4.0 ± 0.4 (*p* < 0.001), respectively. In middle-dose protocols, PCDCT achieved scores of 4.9 ± 0.3, while SDCT and DECT reached scores of 4.5 ± 0.6 (*p* = 0.016) and 3.6 ± 0.5 (*p* < 0.001), respectively. In low-dose protocols, PCDCT scored 4.3 ± 0.5, whereas SDCT and DECT scored 3.7 ± 0.6 (*p* = 0.010) and 2.9 ± 0.6 (*p* < 0.001), respectively. PCDCT also outperformed SDCT in ultra-low-dose settings, with scores of 3.9 ± 0.4 and 2.8 ± 0.4, respectively (*p* < 0.001). Representative images of temporal bone CT acquired with different radiation dose settings are presented in Figs. 5 and 7.

**Fig. 3:**
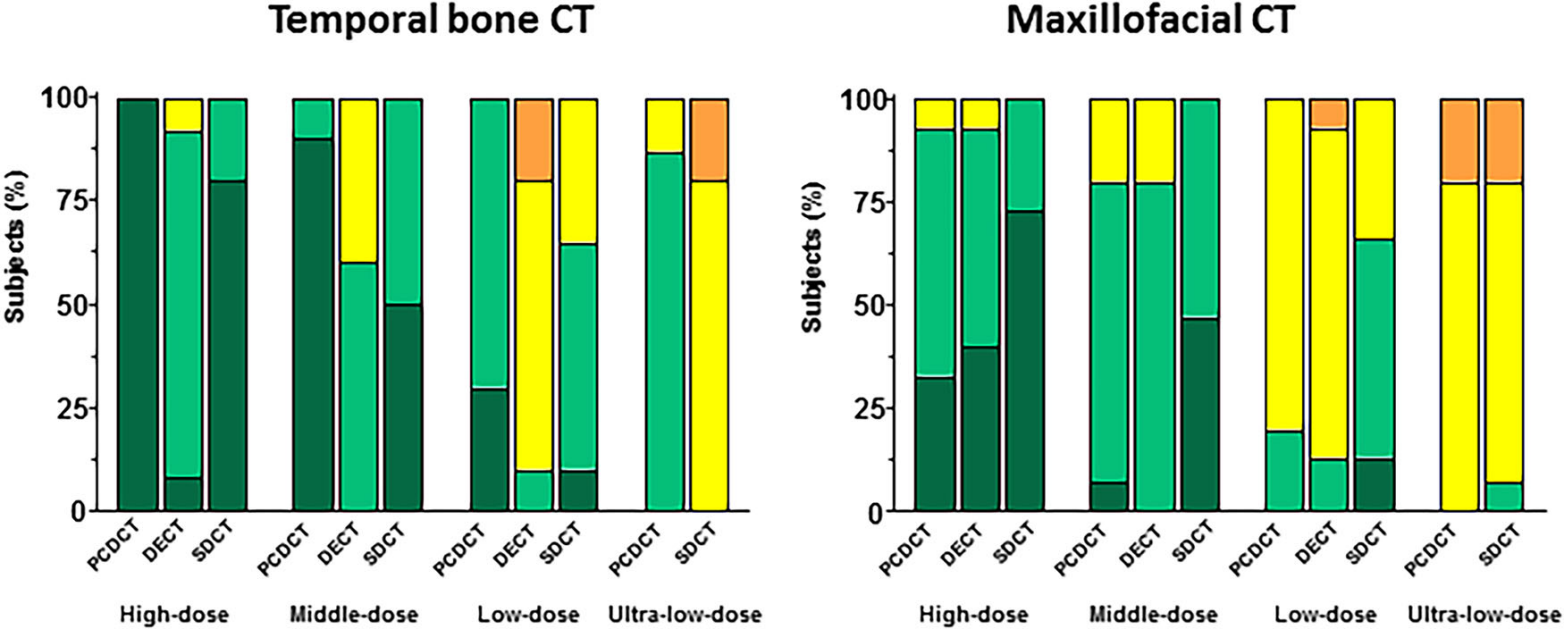
Bar plots of image quality scores for maxillofacial and temporal bone CT using a 5-point Likert grading scale (dark green = excellent; light green = good; yellow = intermediate; orange = poor) obtained in consensus by both readers regarding photon-counting detector CT (PCDCT), dual-source dual-energy CT (DECT) and dual-layer spectral detector CT (SDCT)

Mean values of image quality scores for maxillofacial and temporal bone CT are presented in the Table 1. The frequencies of image quality scores for maxillofacial and temporal bone CT assessed in consensus by both readers are presented in Fig. 3.

### Objective (quantitative) image quality analysis

**Fig. 4:**
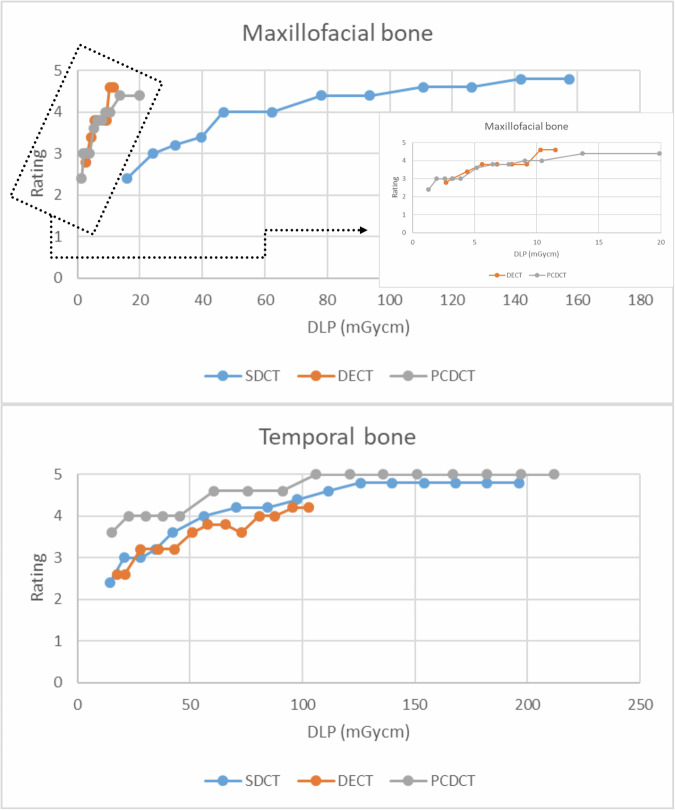
Graphs showing a comparison of DLP and qualitative image ratings between dual-energy, dual-source CT (DECT; orange), spectral detector CT (SDCT; blue) and photon-counting detector CT (PCDCT; gray) in temporal bone and maxillofacial bone

The comparison of quantitative image quality parameters was performed for DECT and PCDCT scanners from the same vendor, in order to exclude bias from vendor-specific reconstruction algorithms and kernel settings. In comparison to DECT, PCD-CT provided significantly better denoising, which remained more stable up to low- and ultra-low-dose scans. PCDCT also demonstrated improved SNR and CNR, which were comparable to those of DECT in high-dose settings. All quantitative parameters of image quality (noise, SNR, CNR) for temporal bone CT are presented graphically in Fig. 6. DLP and CTDI_vol_ for all scans are presented in Fig. 4 and Table 2.

**Fig. 5:**
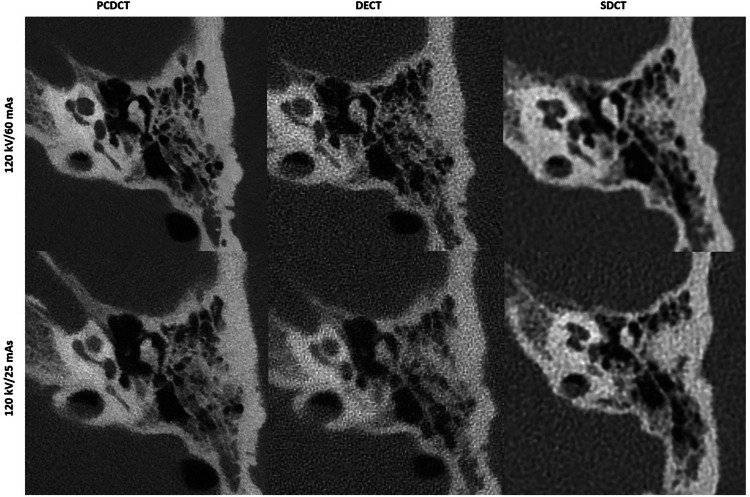
Representative images of temporal bone CT in middle-dose (120 kV/60 mAs) and low-dose (120 kV/25 mAs) scans from photon-counting detector CT (PCDCT), dual-source dual-energy CT (DECT) and dual-layer spectral detector CT (SDCT). PCDCT provided good image quality up to low-dose scans, which is sufficient for clinical use. Improved SNR, CNR and denoising are provided by a PCDCT compared to both EID-based scanners under investigation

**Fig. 6:**
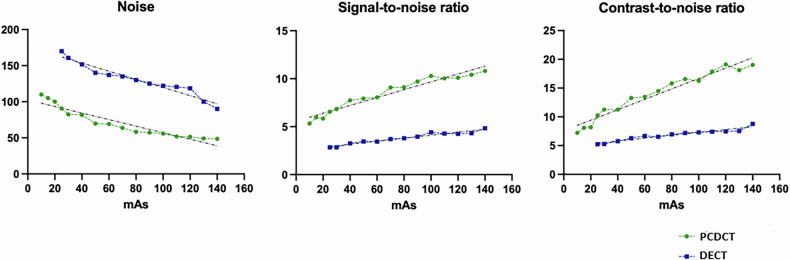
Graphs representing a comparison of different quantitative parameters for image quality assessment (noise, signal-to-noise ratio (SNR) and contrast-to-noise ratio (CNR)) between energy-integrating detector (EID)-based (dual-energy, dual-source; DECT) and photon-counting detector CT (PCDCT) CT in temporal bone CT. Noise, SNR and CNR curves obtained on a clinical DECT (blue line) and a clinical PCDCT (green line)

**Table 2:** DLP and CTDI_vol_ for photon-counting detector CT, dual-source dual-energy CT and dual-layer spectral detector CT in temporal bone and maxillofacial imaging

	Photon-detector	Counting CT	Dual-source integrating	Dual-energy, detector CT	Dual-layer detector CT	Spectral
mAS	CTDI_vol_ (mGy)	DLP (mGycm)	CTDI_vol_ (mGy)	DLP (mGycm)	CTDI_vol_ (mGy)	DLP (mGycm)
**Temporal bone CT**						
140	24	120	18.93	94.65	23.7	118.5
130	22.3	111.5	17.63	88.15	22	110
120	20.5	102.5	16.18	80.9	20.3	101.5
110	18.8	94	14.88	74.4	18.6	93
100	17.1	85.5	13.43	67.15	16.9	84.5
90	15.4	77	12.13	60.65	15.2	76
80	13.7	68.5	10.68	53.4	13.5	67.5
70	12	60	9.38	46.9	11.8	59
60	10.3	51.5	7.93	39.65	10.2	51
50	8.59	42.95	6.63	33.15	8.5	42.5
40	6.87	34.35	5.18	25.9	6.8	34
30	5.15	25.75	3.88	19.4	5.1	25.5
25	4.29	21.45	3.24	16.2	4.2	21
20	3.43	17.15	-	-	3.4	17
15	2.58	12.9	-	-	2.5	12.5
10	1.71	8.55	-	-	1.7	8.5
**Maxillofacial CT**						
100	1.68	16.8	0.81	8.1	11.1	111
90	1.51	15.1	0.73	7.3	10	100
80	1.35	13.5	0.65	6.5	8.9	89
70	1.18	11.8	0.56	5.6	7.8	78
60	1.01	10.1	0.48	4.8	6.6	66
50	0.84	8.4	0.4	4	5.5	55
40	0.67	6.7	0.31	3.1	4.4	44
30	0.5	5	0.23	2.3	3.3	33
25	0.42	4.2	0.19	1.9	2.8	28
20	0.34	3.4	-	-	2.2	22
15	0.25	2.5	-	-	1.7	17
10	0.17	1.7	-	-	1.1	11

*CTDI*_*vol*_ Volume computed tomography dose index, *DLP* Dose-length product

## Discussion

This experimental study aimed to investigate dose-dependent PCDCT image quality in maxillofacial and temporal bone CT and to compare the results with currently available high-end CT systems. The study found that PCDCT provides high image quality (ranging from excellent-to-good) for critical anatomical structures in maxillofacial and temporal bone CT protocols, in some cases superior to EID-based scanners in dose-equivalent scans. Additionally, PCDCT still provided good to sufficient image quality in low- and ultra-low-dose scans for maxillofacial and temporal bone imaging. Results were superior in comparison to EID-based scanners in dose-equivalent scans. Furthermore, PCDCT demonstrated superior denoising and improved SNR/CNR, which remained more stable up to low-dose scans and was superior to that of the EID-based scanners under investigation in dose-equivalent scans.

PCDCT technology has several theoretical intrinsic advantages over EID systems [12, 13]. The main principle of PCDCT systems is that, in contrast to currently used EID-based CT systems, impacting photons are registered quantitatively and qualitatively with a cadmium telluride-based detector and allocated to predetermined energy thresholds and bins. Therefore, PCDCT enables improved spatial resolution, higher energy sensitivity, improved noise reduction continuous spectral imaging, as well as higher dose efficacy, signal- (SNR) and contrast-to-noise ratio (CNR) in low-dose settings compared to the most advanced, currently commercially available state-of-the-art EIDs-based scanners [11, 14, 15].

Increased spatial resolution leads to superior image quality, which is confirmed by the current study results. When comparing image quality of EID- and PCD-based images, the most pronounced differences in subjective image quality scores were seen in temporal bone imaging for all doses and when comparing dose-equivalent scans for the maxillofacial region. This holds true even when comparing low-dose PCDCT images with mid-dose and high-dose EIDCT images (see also Figs. 2, 5). As the ultra-high resolution mode is only available for PCDCT, this mode was not additionally investigated to allow for comparison between all scanners. For maxillofacial CT, similar subjective image quality scores were achieved by PCDCT and DECT in high-, middle-, and low-dose settings. This, however, was lower than that of SDCT in high- and low-dose scans. Nevertheless, superior subjective image quality of SDCT compared to PCDCT and DECT was achieved by approximately ten-fold higher radiation doses in all scans, as there was no tin filtration available in the SDCT scanner. When comparing dose-equivalent scans, PCDCT outperformed SDCT and DECT in this study. Regarding dose-equivalent scans, PCDCT provides higher image quality also for maxillofacial CT compared to SDCT (*e.g.*, high-dose scans of PCDCT *versus* low-dose scans of SDCT by comparable radiation exposure, please also see Table 1 and Fig. 4). Moreover, subjective image quality of PCDCT in both maxillofacial (good-to-sufficient) and temporal bone (excellent-to-good) imaging remained stable up to low- and ultra-low-dose scans.

No differences were found in image quality for the maxillofacial region in ultra-low-dose settings between PCDCT and SDCT, again with significantly reduced radiation dose for PCDCT (see Fig. 4).

Continuous spectral imaging is only possible using SDCT and PCDCT, leading to numerous advantages, such as the possibility to retrospectively reconstruct virtual monoenergetic images, iodine maps, virtual non-calcium images and virtual non-contrast images [11, 16].

**Fig. 7:**
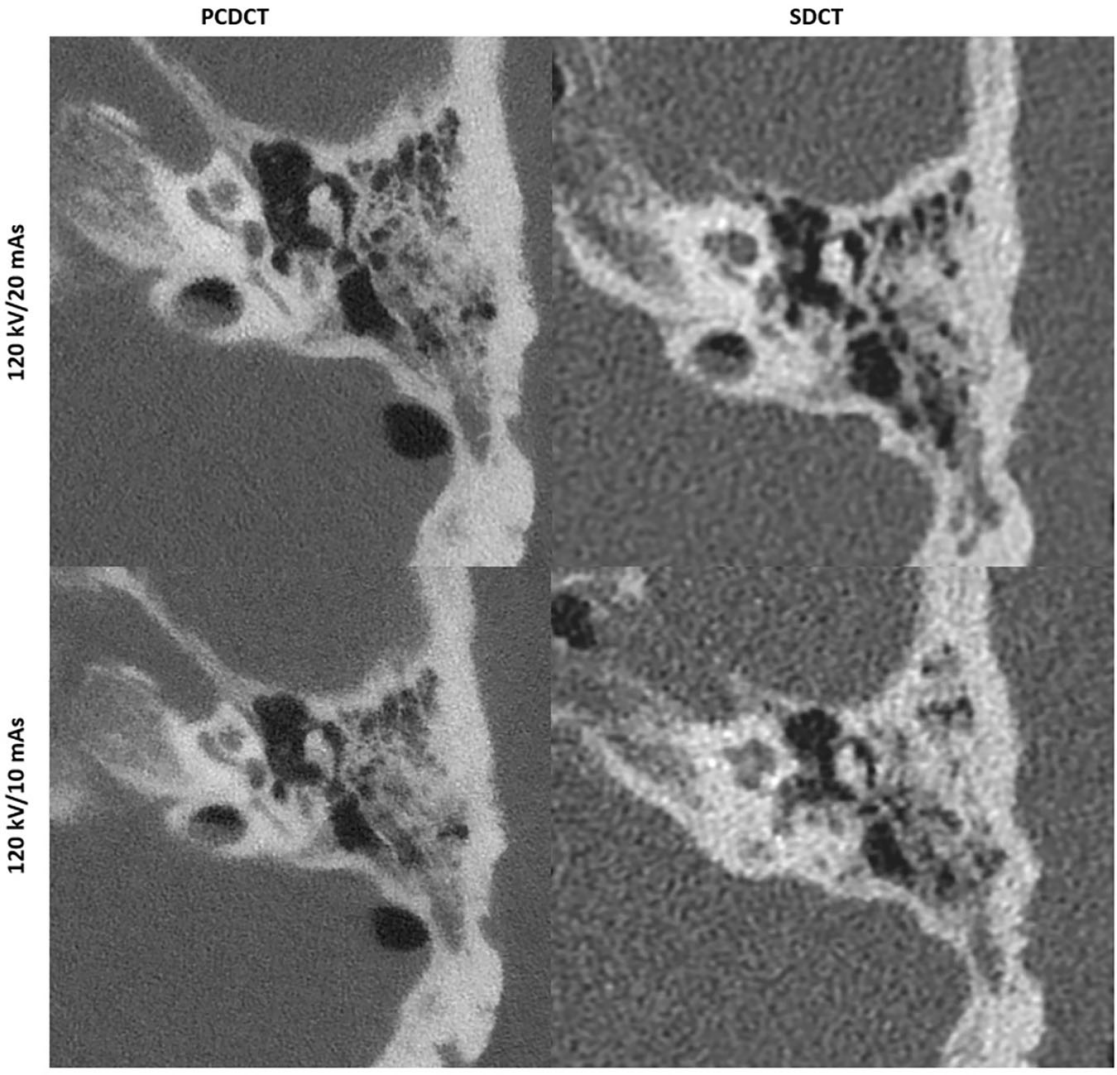
Representative images of ultra-low-dose CT scans of the temporal bone on photon-counting detector CT (PCDCT) and dual-layer spectral detector CT (SDCT)

Another potential advantage of PCDCT is higher energy sensitivity, reflected in superior denoising and improved CNR/SNR. Noise levels and image quality were superior for PCDCT in comparison to dose-equivalent scans from the other investigated high-end scanners. These findings are in line with previous experimental and preclinical data demonstrating considerably higher SNR and CNR and superior denoising by PCDCT [7, 11, 17], reflecting less susceptibility to background noise during image acquisition (see also Fig. 6). These findings suggest that the advantages of PCDCT are most evident in low- and ultra-low-dose settings (Fig. 7). Inter-vendor (SDCT and PCDCT/DECT) comparison of quantitative image quality parameters was not performed, as specific reconstruction algorithms and reconstruction kernels differ significantly between vendors (*i.e.*, Philips Healthcare and Siemens Healthineers) prohibiting reliable comparison of quantitative parameters.

The reduced radiation exposures of PCDCT and DECT in maxillofacial CT in comparison to SDCT are a result of x-ray beam hardening indebted to tin filter employment, SDCT does not enable this feature as shown in Fig. 4. Therefore, a direct comparison of subjective image quality scores is difficult as radiation exposure has a direct influence on image quality [18]. For the temporal bone, no tin filter was employed; in this experimental setup, PCDCT showed its advantages of higher image quality with comparable radiation dose.

Previous studies investigating maxillofacial and temporal image quality of PCDCT lack a comparison to SDCT. The current findings align with the results reported by Grunz et al, who demonstrated that a clinical PCDCT system exhibits superior image quality compared to DECT in both maxillofacial and temporal bone imaging. [6, 7]. Low- and ultra-low-dose imaging is of clinical importance if drastic radiation dose reduction can be achieved while maintaining sufficient diagnostic quality.

Neither DSCT nor SDCT enables Ultra-high resolution mode; therefore, it was not investigated in the current comparative study.

In a previous PCD-CT study on sinus and temporal bone image quality performed on phantoms and cadavers, ultra-high-resolution mode was combined with a tin filter; compared to a second-generation dual-source CT system, improved image quality and reduced radiation dose were found [17]. Another study on a cadaveric temporal bone by Leng et al on a research PCDCT suggested 29% noise reduction, whereas Zhou et al reported up to 40% noise reduction [10, 19, 20]. Our study confirmed a noise reduction of up to 40% for a clinical PCDCT.

Besides scanner hardware, the image reconstruction influences the final image resolution. Typically, filtered backprojection algorithms serve as a basis reconstruction step on modern CT scanners, iterative algorithms complement these. During the filtering step of a filtered backprojection, the measured and preprocessed projection data are convolved with a filter kernel, which can differ depending on the manufacturer and also the device. By modifying this filter kernel, image quality can be influenced. It is difficult to compare different reconstruction algorithms as they differ in the three investigated scanners. However, a non-filtered back projection is often not possible. As reconstruction algorithms evolve and there are considerable differences between the vendors, especially with the nowadays even AI-based reconstructions, there is a substantial impact on image appearance and quality. The scans performed in this study used iterative reconstruction algorithms and reconstruction kernels based on the vendor recommendations, using the settings configured in the specific program, which limits comparability considerably. In future studies, this limitation could be addressed by working with non-filtered back projection; however, this was beyond the scope of the study. Examinations were performed on a single cadaveric specimen scanned multiple times on the same day within 1.5 h, which may limit the transferability of the current results. It is important to note that cadaveric tissue has altered characteristics as a result of tissue necrosis, such as water retention, affecting radiation attenuation and image quality. However, conducting multiple scans on human subjects for the purpose of dose-dependent quality assessment as well as inter-vendor comparisons raises ethical concerns.

Furthermore, recent studies conducted on patients found similar results [8, 9]. However, further prospective studies in patients using different low- and ultra-low-dose protocols are required to establish the results of this study in routine clinical practice. Another limitation is that a comparison between scanners from different vendors (Siemens *versus* Philips) is not entirely possible due to vendor-specific technical aspects/settings (*e.g.*, tin filtration implemented in Siemens scanners, noise texture). Comparison of quantitative markers of image quality is not routinely possible due to the non-uniformity of reconstruction kernels. To address this limitation, subjective, radiation dose-adapted image quality was assessed.

In conclusion, PCDCT performs in some cases better than modern EID-CT and SDCT for the assessment of maxillofacial and temporal bone CT in image quality and required radiation dose. Our study results suggest that the broad implementation of PCD-based CT systems with further optimization of scan protocols will improve image quality with the prospect of reduced radiation exposure in the general population. However, PCDCT is, up to now, an expensive modality that is not affordable in most clinical settings and most countries. Therefore, overall, SDCT remains the clinically widespread “standard” that needs to be surpassed.

## Supplementary information

ELECTRONIC SUPPLEMENTARY MATERIAL

## Data Availability

The datasets generated or analyzed during this study are available from the authors upon reasonable request.
