# Supplementary material for: Photon-counting CT in maxillofacial and temporal bone CT—a comparative analysis of image quality and dose with high-end energy-integrating CT systems
Source: Eur Radiol Exp. 2025 Aug 15;9:77. doi: 10.1186/s41747-025-00618-6 (PMC12356771; doi:10.1186/s41747-025-00618-6)
Supplement: Supplementary file 1 — ELECTRONIC SUPPLEMENTARY MATERIAL [file 41747_2025_618_MOESM1_ESM.pdf]

Photon counting CT in maxillofacial and temporal bone CT - a comparative analysis of image quality and dose with high end energy integrating CT systems

ELECTRONIC SUPPLEMENTARY MATERIAL

Eur Radiol Exp (2025) Layer YC, Mesropyan N, Isaak A, et al.

Temporal bone								
Photon counting detector CT	kV	mAS	overall	facial nerve canal	cochlea	tympanic tegmen	malleus	Mean
	120	140	5	5	5	5	5	5
	120	130	5	5	5	5	5	5
	120	120	5	5	5	5	5	5
	120	110	5	5	5	5	5	5
	120	100	5	5	5	5	5	5
	120	90	5	5	5	5	5	5
	120	80	5	5	5	5	5	5
	120	70	5	5	5	5	5	5
	120	60	4	5	5	4	5	4.6
	120	50	4	5	5	4	5	4.6
	120	40	4	5	5	4	5	4.6
	120	30	4	4	4	4	4	4
	120	25	4	4	4	4	4	4
	120	20	4	4	4	4	4	4
	120	15	4	4	4	4	4	4
	120	10	4	4	4	3	3	3.6
Dual-source dual-energy CT		mAS	overall	facial nerve canal	cochlea	tympanic tegmen	malleus	Mean
	120	140	4	5	4	4	4	4.2
	120	130	4	5	4	4	4	4.2
	120	120	4	4	4	4	4	4
	120	110	4	4	4	4	4	4
	120	100	4	4	4	3	3	3.6
	120	90	4	4	4	3	4	3.8
	120	80	4	4	4	3	4	3.8
	120	70	4	4	4	3	3	3.6
	120	60	3	3	4	3	3	3.2
	120	50	3	3	4	3	3	3.2
	120	40	3	3	4	3	3	3.2
	120	30	3	2	3	3	2	2.6
	120	25	3	2	3	3	2	2.6
Dual-layer spectral detector CT		mAS	overall	facial nerve canal	cochlea	tympanic tegmen	malleus	Mean
	120	141	4	5	5	5	5	4.8
	120	130	4	5	5	5	5	4.8
	120	120	4	5	5	5	5	4.8
	120	109	4	5	5	5	5	4.8
	120	100	4	5	5	5	5	4.8
	120	91	4	5	5	5	5	4.8
	120	80	4	5	4	5	5	4.6
	120	70	4	5	4	5	4	4.4
	120	59	4	4	5	4	4	4.2
	120	50	4	4	5	4	4	4.2
	120	41	4	4	5	4	3	4
	120	30	4	4	4	3	3	3.6
	120	24	3	3	4	3	3	3.2
	120	20	3	3	3	3	3	3
	120	15	3	3	3	3	3	3
	120	10	3	3	2	2	2	2.4

Maxillofacial

Photon counting detector CT	kV	mAs	Overall IQ	Uncinate process of the etmoid bone	Nasolacrimal duct	Cribriform plate	Ostium of maxillar sinus	Mean
	100	100	5	4	4	4	5	4.4
	100	90	5	4	4	4	5	4.4
	100	80	4	4	4	3	5	4
	100	70	4	4	4	3	5	4
	100	60	4	4	4	3	4	3.8
	100	50	4	4	4	3	4	3.8
	100	40	4	4	4	3	3	3.6
	100	30	3	3	3	3	3	3
	100	25	3	3	3	3	3	3
	100	20	3	3	3	3	3	3
	100	15	3	3	3	3	3	3
	100	10	3	3	2	2	2	2.4

Dual-source dual-energy CT	kV	mAs	Overall IQ	Uncinate process of the etmoid bone	Nasolacrimal duct	Cribriform plate	Ostium of maxillar sinus	Mean
	100	100	5	5	4	4	5	4.6
	100	90	5	5	4	4	5	4.6
	100	80	4	4	4	3	4	3.8
	100	70	4	4	4	3	4	3.8
	100	60	4	4	4	3	4	3.8
	100	50	4	4	4	3	4	3.8
	100	40	4	3	4	3	3	3.4
	100	30	3	3	3	3	3	3
	100	25	3	3	2	3	3	2.8

Dual-layer spectral detector CT	kV	mAs	Overall IQ	Uncinate process of the etmoid bone	Nasolacrimal duct	Cribriform plate	Ostium of maxillar sinus	Mean
	100	100	5	5	5	4	5	4.8
	100	91	5	5	5	4	5	4.8
	100	79	5	5	4	4	5	4.6
	100	70	5	5	4	4	5	4.6
	100	60	5	5	4	4	4	4.4
	100	50	5	5	4	4	4	4.4
	100	40	5	4	4	3	4	4
	100	31	5	4	4	3	4	4
	100	25	4	4	3	3	3	3.4
	100	19	4	3	3	3	3	3.2
	100	15	3	3	3	3	3	3
	100	10	3	3	2	2	2	2.4
